# 2016年胸外科医师年会早期肺癌特邀专题编者按

**DOI:** 10.3779/j.issn.1009-3419.2016.06.04

**Published:** 2016-06-20

**Authors:** 克能 陈

**Affiliations:** 北京大学肿瘤医院 Beijing Cancer Hospital

在西方国家肺癌发病率有所下降的今天，我国肺癌的发生仍呈现出急剧上升的态势。可喜的是，近年来伴随着国家经济水平的发展、国民健康意识的觉醒、胸部计算机断层扫描（computed tomography, CT）引入肺癌普查临床实践的不断开展，临床早期肺癌的检出率越来越高。尤其合并重症及高龄的病人大量出现在临床。毋庸置疑，早期肺癌的治疗应以外科切除为主，然而，临床已经发现其中一些是多发性的，一些是经年不变的惰性肿瘤。对这些特殊肺癌究竟如何处置，首先要有一支专业齐全、素质极高的多学科治疗（multiple disciplinary team, MDT）团队，包括优秀的影像专家、病理专家、重症专家、麻醉专家等构成的MDT，为这一特殊类型肺癌的诊疗，就何时要做、如何做、做什么等问题作出多学科背景下的顶层设计。这样即便最终选择了手术，也是在多学科、跨学科背景协作下的手术治疗，避免了一部分无谓的手术与过度治疗。对部分高危又必须治疗的患者也可选择立体定向放射治疗（stereotactic body radiation therapy, SBRT）作为手术的替代治疗方法，或甚至做出不做任何治疗仅做临床观察的理性决定，最大程度地避免了医源性伤害所引起的不必要的死亡率和并发症率。当然，我们也期待在不远的将来发现可操作性强的、用于预测惰性肿瘤的指标。有关早期肺癌的这些分子水平、病理诊断、影像表现、临床转归及诊断治疗等各方面的新知识、新理论和新技术，从根本上影响甚至改变了早期肺癌，尤其是与外科诊疗相关的临床实践。然而，这些理论、知识还较为零散，不便于外科医师，尤其是基层工作者所查找。

因此，借胸外科医师分会2016年年会召开之际，我们特别邀请了活跃在我国肺癌临床一线的院士、知名专家撰写了这期早期肺癌相关的教育性文献，作为本届年会教育集的一部分内容奉献给大家，承蒙《中国肺癌杂志》厚爱，得以以正式刊物出版，希望对广大基层胸外科医师有所帮助，同时对各位专家辛苦的读书、总结，并无私与大家分享自己对早期肺癌诊断治疗的心得表示衷心感谢。但由于时间仓促、认识水平有限，难免存在漏洞，也未能将更多能人志士对早期肺癌的心得体会全部纳入其中，在此一并致歉。

